# Ovarian carcinoma metastasis manifesting as periumbilical nodules^⋆^

**DOI:** 10.1016/j.abd.2021.01.009

**Published:** 2022-09-29

**Authors:** Ivonne Dannesy Rodriguez Hernandez, Patricia de Franco Marques Ferreira, Paula Dadalti Granja, Mayra Carrijo Rochael

**Affiliations:** a Department of Dermatology, Hospital Universitário Antonio Pedro, Universidade Federal Fluminense, Niteroi, RJ, Brazil; b Section of Dermatopathology, Department of Pathology, Hospital Universitário Antonio Pedro, Universidade Federal Fluminense, Niteroi, RJ, Brazil

Dear Editor,

The skin can signal internal diseases such as visceral neoplasms. Any tumor has the potential to cause skin metastases, which can occur via lymphatic, hematogenous routes, contiguity or iatrogenic implantation. Subcutaneous nodules can have inflammatory, infectious or neoplastic etiologies. Skin metastases occur in 1% to 9% of malignancies, and about 10% affect the umbilical region.^1^, ^2^

Metastasis to the umbilical region has been described as Sister Mary-Joseph's nodule. It is a painless, palpable lump that varies in color from violaceous to reddish-brown and can resemble a vascular structure.^2^ The main origin is the abdominal and pelvic viscera, with the gastrointestinal tract being the most common site in males and the gynecological tract in females, particularly ovarian tumors. Adenocarcinoma is the most frequent histological type comprising 75% of cases.^3^

A 45-year-old female patient presented with painless nodular lesions in the umbilical and periumbilical region for 6 months, associated with weight loss and abdominal pain. Clinically, she was pale, emaciated, showing a globular and ascitic abdomen, an abdominal mass in the pelvic region, and purplish-brown nodules with an ulceronecrotic surface and hardened consistency in the umbilicus and periumbilical region (Fig. 1). The abdominal ultrasonography disclosed an enlarged left ovary, and nodules suggestive of peritoneal and liver implants (Fig. 2). Histopathology of one of the periumbilical lesions showed neoplastic tissue, consisting of atypical glands covered by high cylindrical epithelium with basal nuclei, associated with mucinous material, compatible with metastatic mucinous adenocarcinoma (Fig. 3). Immunohistochemistry showed strong and diffuse positivity with CK20, CEA and p16 antibodies; focal positivity with CA125 and immunonegativity for estrogen receptors and CK7. The CK20 positive and CK7 negative histochemical profile favors the intestinal variant of an metastasis, that is a metastatic ovarian malignancy. After the diagnosis, the patient was referred to the oncology service but died after one month.

**Fig. 1:**
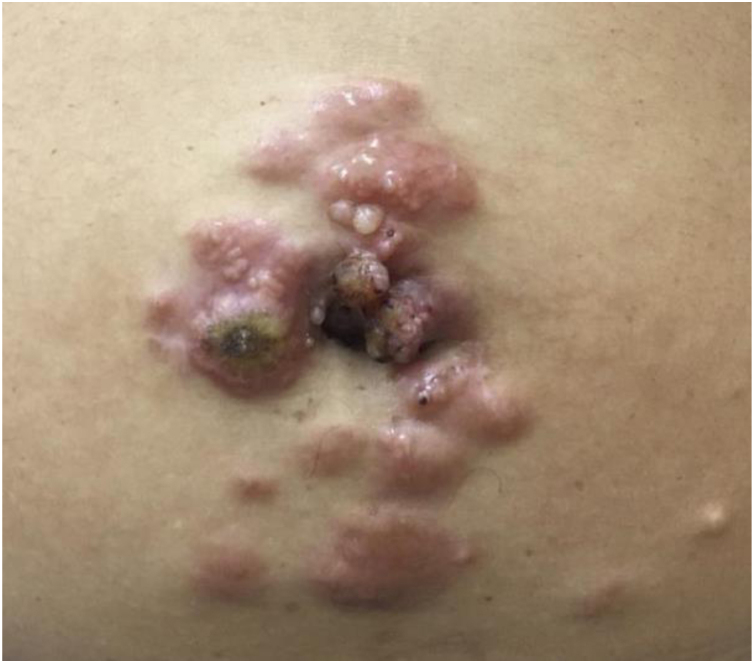
Multiple purplish-brown nodules with an ulceronecrotic surface in the umbilicus and periumbilical region.

**Fig. 2:**
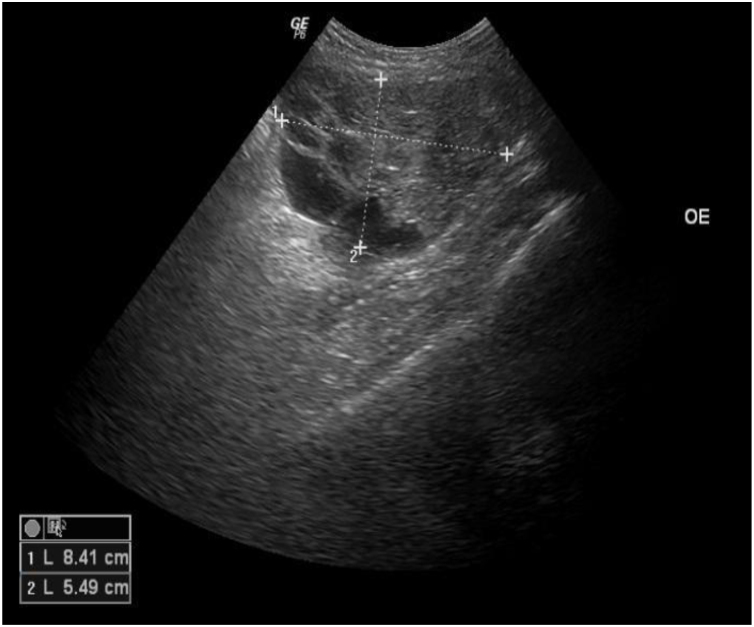
Left ovary enlargement (8 × 5 cm) with small cystic formations.

**Fig. 3:**
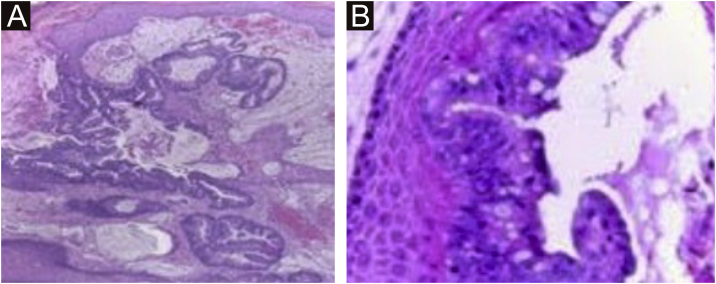
Neoplastic tissue consisting of atypical glands covered by high cylindrical epithelium with basal nuclei associated with mucinous material; diagnosis: metastatic mucinous adenocarcinoma. (Hematoxylin & eosin, ×40). In the detail, high glandular cells and goblet cells over stratified squamous epithelium (Hematoxylin & eosin, ×400).

It is essential to know the causes of umbilical nodules, since this cutaneous manifestation can represent an opportunity to make the diagnosis of the primary lesion. Some differential diagnoses are cutaneous endometriosis, pyogenic granuloma, melanoma, squamous cell/basal cell carcinoma, and umbilical hernia.^3^ Biopsy is a low-risk procedure, considering its high yield and allows an early diagnosis, aiming to reduce morbidity and mortality. Histopathology of the lesion complemented by immunohistochemistry should be performed whenever possible since it can determine the tumor origin or guide the clinical and imaging assessment of the primary site. Metastases with glandular microscopic characteristics require the differential diagnosis between several primary neoplasms. The CK7- CK20+, CK7+ CK20+, and CK7- CK20- panels generally indicate tumors that include the bladder, gastrointestinal tract, pancreas and rare ovarian tumors.

Findings similar to those of the present case (CK7+ CK20-) correspond to primitive tumors that can be located in the breast, lung, thyroid, and gynecological tumors. Specific antibodies were used, excluding adenocarcinoma of other origins and indicating the ovary as the strongest possibility of the primary origin of the umbilical metastases. Finally, it is important to note that in the presence of umbilical nodules, the possibility of cutaneous metastases should be considered.^4^

## Financial support

None declared.

## Authors' contributions

Ivonne Dannesy Rodriguez Hernandez: Approval of the final version of the manuscript; design and planning of the study; drafting and editing of the manuscript; intellectual participation in the propaedeutic and/or therapeutic conduct of the studied cases; critical review of the literature; critical review of the manuscript

Patricia de Franco Marques Ferreira: Approval of the final version of the manuscript; design and planning of the study; drafting and editing of the manuscript; intellectual participation in the propaedeutic and/or therapeutic conduct of the studied cases; critical review of the literature; critical review of the manuscript.

Paula Dadalti Granja: Approval of the final version of the manuscript; design and planning of the study; effective participation in research orientation; intellectual participation in the propaedeutic and/or therapeutic conduct of the studied cases; critical review of the literature; critical review of the manuscript.

Mayra Carrijo Rochael: Approval of the final version of the manuscript; drafting and editing of the manuscript; effective participation in research orientation; intellectual participation in the propaedeutic and/or therapeutic conduct of the studied cases; critical review of the literature; critical review of the manuscript.

## Conflicts of interest

None declared.
